# Splicing regulation and intron evolution in the short-intron ciliate model of endosymbiosis *Paramecium bursaria*

**DOI:** 10.1093/nar/gkag063

**Published:** 2026-02-02

**Authors:** Thi Ngan Giang Nguyen, Md Mostafa Kamal, Chien-Ling Lin, Jun-Yi Leu

**Affiliations:** Institute of Molecular Biology, Academia Sinica, Taipei 11529, Taiwan; Genome and Systems Biology Degree Program, National Taiwan University and Academia Sinica, Taipei 106, Taiwan; Institute of Molecular Biology, Academia Sinica, Taipei 11529, Taiwan; Institute of Molecular Biology, Academia Sinica, Taipei 11529, Taiwan; Institute of Molecular Biology, Academia Sinica, Taipei 11529, Taiwan

## Abstract

The integration of symbionts into host cells during endosymbiosis significantly alters gene expression and cell physiology. Though alternative splicing facilitates cellular adaptation through rapid modulation of gene expression and protein isoform diversity, its regulatory role during endosymbiosis remains poorly understood. *Paramecium bursaria*, which harbors hundreds of *Chlorella variabilis* algae within its cytoplasm, offers a powerful model to study splicing during endosymbiosis, especially given its exceptionally short introns (median ∼24 nt). Using time-course RNA sequencing of symbiotic and aposymbiotic cells, we found that splicing, especially of 5′ proximal introns, enhances gene expression. Moreover, we identified 883 genes with differentially spliced introns, particularly enriched in transmembrane transporters essential for establishing nutrient exchange between a host cell and algal symbionts. Splicing regulation correlated with expression changes in conserved spliceosome components, implicating that these factors act as splicing enhancers or repressors during symbiosis. By exploring intron orthology across ciliates, we found that conserved introns exhibited more efficient splicing, characterized by lower GC content and uniform length, suggesting that intron evolution favors features that optimize expression. Our study reveals how splicing contributes to host adaptation during endosymbiosis and highlights the evolutionary dynamics of short introns in eukaryotes.

## Graphical Abstract

**Graphical Abstract:**
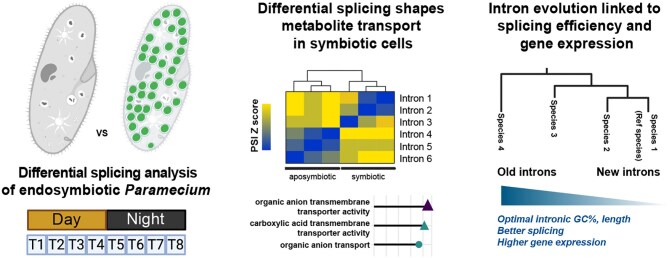

## Introduction

RNA splicing is a crucial post-transcriptional process in eukaryotes, involving exon ligation and intron removal. The U2-dependent splicing pathway is the primary mechanism for intron removal in eukaryotic pre-mRNA, which proceeds through stepwise assembly of the spliceosome on splice junctions [1]. The major components of the U2-dependent spliceosome include five essential small nuclear ribonucleoproteins (i.e. the U1, U2, U4/U6, and U5 snRNPs), which are comprised of associated U-rich small nuclear RNAs (UsnRNAs) and other associated proteins. In human cells, the 5′ splice site (5′SS), branch point site (BPS), and 3′ splice site (3′SS) are recognized by U1 snRNP, SF1 (Splicing Factor 1), and U2AF1 (U2 Small Nuclear RNA Auxiliary Factor 1). SF1 is then displaced by U2 snRNP, followed by recruitment of the U4/U6.U5 tri-snRNP. Subsequent rearrangements and release of U1 and U4 yield the catalytically active complex, which executes the first splicing step. Further remodeling facilitates exon ligation and spliceosome recycling.

Beyond the core spliceosome, splicing is tightly regulated by *cis-*acting regulatory elements (enhancers and silencers) and *trans*-acting splicing factors [2, 3, 4]. Enhancer elements are typically bound by splicing activators such as serine/arginine-rich (SR) proteins, which promote splicing. In contrast, silencer elements are bound by repressor proteins such as heterogeneous nuclear ribonucleoproteins (hnRNPs), leading to splicing inhibition [5–7].

Alternative splicing (AS) is a crucial aspect of the regulation of various biological processes, including cell identity and circadian rhythms [8–11]. AS diversifies gene expression by generating multiple isoforms through exon skipping, alternative splice sites, mutually exclusive exons, or intron retention [12]. When AS introduces premature termination codons (PTCs), it activates the nonsense-mediated decay (NMD) pathway, leading to messenger RNA (mRNA) degradation [13]. The NMD pathway has been shown to regulate transcript levels and ensure splicing fidelity [14–16]. In contrast, in-frame AS events result in changes in protein sequences and structures, thereby altering protein activity or cellular localization [17, 18].

Endosymbiosis involves the integration of symbionts into host cells, forming a mutually beneficial relationship. Endosymbionts confer novel phenotypic traits on the host, enabling its exploitation of more diverse environmental resources, while relying on the host for essential nutrients [19]. The “Endosymbiosis Theory” explains the origin of mitochondria and chloroplasts through integration of ancestral prokaryotes into early eukaryotic cells [19, 20]. This integration can fundamentally alter host cell biology, such as by transforming photosynthetic cyanobacteria into chloroplasts within plant cells or driving the development of specialized organelles to accommodate endosymbionts [19, 21]. To maintain such symbiotic relationships, both host cells and endosymbionts must undergo extensive physiological and genomic adaptations, including horizontal gene transfer and evolution of nutrient and resource exchange mechanisms [20, 22–25].

*Paramecium bursaria* serves as an excellent model for studying endosymbiosis. *Paramecium bursaria* stably hosts hundreds of algal cells shielded from lysosome digestion by the perialgal vacuole (PV) beneath the host cytoplasmic membrane [26–28]. Symbiotic cells typically display increased size and growth rate, and reduced numbers of mitochondria near PVs [27, 29]. Transcriptomic comparisons between symbiotic and aposymbiotic *P. bursaria* revealed downregulation of redox, aminotransferase, and ribosomal proteins and upregulation of Hsp70, the Myb transcription factor, and histidine kinase pathway genes in the symbiont-bearing host cells [30]. While transcriptional regulation has been studied [30–33], the functional role of AS regulation in endosymbiosis remains unexplored.

Introns exert a crucial role in various evolutionary processes due to their links to regulating adaptive gene expression. Intron gain occurs through several mechanisms, including transposon or retroelement insertion, reverse splicing of intron RNA, tandem duplications that generate new splice sites, DNA double-strand break repair, and exon “intronization” caused by mutations that create novel splice junctions [34–36]. These events expand gene length and complexity, provide opportunities for alternative splicing, and facilitate exon shuffling, thereby driving protein and regulatory innovation. Newly gained introns can also carry enhancers or other *cis-*elements, contributing to intron-mediated enhancement of gene expression. Intron size varies widely across species, ranging from 15 nucleotides (nt) in the ciliate *Stentor coeruleus* to over 1 million nt in humans [37, 38]. While vertebrates exhibit a bimodal distribution of intron lengths [37], ciliates such as *Paramecium* and *Tetrahymena* predominantly harbor extremely short introns (<100 nt). Despite the brevity, intron splicing in ciliates still plays a significant role in gene regulation [39–41]. Thus, comparative studies of intron evolution across ciliates can offer valuable insights into the evolutionary history and functional adaptation of short introns, as well as their implications for transcriptomic regulation.

In this study, we examined time-course RNA sequencing data from both symbiotic (symbiont-bearing) and aposymbiotic (symbiont-free) *P. bursaria* cells to quantify AS across stages and uncover its relationship with gene expression. Differential intron retention analysis revealed specific splicing regulation, particularly in genes associated with transmembrane transporter activity, a function closely linked to nutrient exchange during endosymbiosis. Comparative intron orthology across *Paramecium* species further demonstrated that intron length and GC content have undergone evolutionary refinement to optimize splicing efficiency. Collectively, our findings highlight how intron architecture has been shaped to enhance splicing precision and metabolic fitness in the context of endosymbiotic adaptation.

## Materials and methods

### *Paramecium bursaria* strain and culture conditions

We used the *P. bursaria* DK2 strain, which harbors the endogenous endosymbiont *Chlorella variabilis*. DK2 is an offspring of the Dd1 and KM2 strains, and details of the process for creating DK2 have been described previously [42]. *Paramecium bursaria* cells were cultured on 2.5% Boston lettuce in Dryl’s solution medium and fed with *Klebsiella pneumoniae* (NBRC 100048 strain). Cell cultures were maintained at 23°C with a 12-h light/dark cycle. Bacteria-containing lettuce media was refreshed every 3 days. To produce aposymbiotic (white) *Paramecium* strains, we used cycloheximide (10 μg/ml) to treat symbiotic (green) cells, as described previously [43].

### Genome annotation file and intron annotation

Like other ciliate species, *P. bursaria* possesses a diploid genome displaying allele duplication. The genome annotation file (GTF) was assembled according to a method described previously [42]. We separated the diploid genome annotation into two haplotypes and identified 14,920 functional genes as representatives. Intron positions were determined using an in-house R script by extracting the sequences located between two consecutive exons.

### RNA extraction and RNA sequencing

For RNA sequencing, *P. bursaria* green (symbiotic) and white (aposymbiotic) cells were harvested every 3 h (2 AM, 5 AM, 8 AM, 11 AM, 2 PM, 5 PM, 8 PM, 11 PM) for RNA extraction, with a total of three replicates for each of 16 samples.

For RNA extraction, ~10^5^  *P. bursaria* cells were collected in early stationary phase, washed twice with 1× Dryl’s buffer, and concentrated using an 11-μm-pore-size nylon membrane. An RNeasy Mini Kit (Cat No. 74106, QIAGEN) and TRI Reagent (T9424, Sigma–Aldrich) were used to extract total RNA as described previously [44]. Strand-specific RNA-seq libraries were generated using the SureSelect Strand-Specific RNA Library Preparation Kit for Illumina (Agilent Technologies, Santa Clara, CA, USA) according to the supplier’s protocol. Sequencing was performed by Welgene Biotech Co., Ltd. (Taiwan) on a NovaSeq 6000 platform (Illumina, San Diego, CA, USA), producing 150-bp paired-end reads for three independent biological replicates in each condition (symbiotic and aposymbiotic).

The adapters and low-quality reads were trimmed using fastp for paired-end reads with the following parameters: cut_front_window_size = 3; cut_tail_window_size = 3; cut_right_window_size = 4; cut_right_mean_quality = 30; length_required = 36 [45]. Then, the trimmed reads were aligned to the *P. bursaria* genome [42] using STAR 2.7.11a to create BAM files with the following criteria: twopassMode = Basic; overSJfilterOverhangMin = 7 5 5 5; outSAMstrandField = intronMotif; alignIntronMin = 10; alignIntronMax = 10000; alignEndsType = EndToEnd; outSAMmapqUnique = 3; outSAMtype = BAM SortedByCoordinate [46].

### Gene expression analysis

Salmon 0.13.1 was used to calculate raw gene read counts for each sample using quality-controlled FASTQ files, option gcBias, and numBoostraps = 200 [47]. The tximport software was used to import raw gene counts from Salmon into R, which were then utilized in DESeq2 to normalize gene expression [48, 49]. The differentially expressed genes (DEGs) were then analyzed using DESeq2 by computing the log_2_ fold-change (Log_2_FC) of genes between conditions and the false discovery rate (FDR) of the difference in expression level significance [49]. For DESeq2 to compute, the number of normalized reads for each gene must be >10 in at least three replicates. If Log_2_FC $ \ge $ 1 and FDR $ \le $ 0.05, then a given gene was defined as differentially expressed between two groups under consideration.

### Analyses of alternative splicing events, intron retention rate (PSI), & differential spliced intron clustering

rMATs (replicate multivariate analysis of transcript splicing) was used to quantify alternative splicing (AS) events, including exon skipping, intron retention, mutually exclusive exons, alternative 3′ splice sites, and alternative 5′ splice sites [50]. Aligned BAM files from STAR were used as input for rMATs with options novelSS, allow-clipping, and mil (minimum intron size) = 10 to identify the AS events between symbiotic and aposymbiotic cells. We filtered differential splicing efficiency according to |∆PSI| ≥ 0.1, junction read count (inclusion read + exclusion read) > 10, and FDR < 0.05 to define differentially spliced introns (DSIs) between groups.

The individual intron retention rate was determined using SQUID (https://github.com/Xinglab/SQUID). Percent splice in (PSI) was calculated using the formula: total inclusion reads divided by total number of junction reads (Supplementary Fig. S3) [42].


\begin{eqnarray*}
{\mathrm{PSI}} = {\mathrm{\ }}\frac{{{\mathrm{Inclusion\ read}}.0.5}}{{{\mathrm{Inclusion\ read}}.\ 0.5{\mathrm{\ }} + {\mathrm{Spliced\ read}}}}.
\end{eqnarray*}

The PSI of introns was only included in our analysis if the number of junction read counts was 10 or greater, and the standard deviation for PSI for three replicates was < 0.1 in all of the replicates.

The PSI of each alternatively spliced intron between symbiotic and aposymbiotic cells was compared in a pairwise fashion at each time point using SQUID. Introns displaying statistical significance at |∆PSI| > 0.1 and FDR < 0.05 between symbiotic and aposymbiotic cells were selected for further investigation.

### GO term enrichment analysis

Protein functions for DK2 genes were predicted using multiple tools following the comprehensive approach described previously [44]. InterProScan v5.40-77 with the --goterms option was used to assign Gene Ontology (GO) terms to proteins. Functional annotation was expanded by inferring protein-protein interactions using DScript v0.2.2 [51] with the trained model topsy_turvy_v1.sav. An interaction-probability threshold of 0.92 was chosen to match the density of predicted positives observed in *Saccharomyces cerevisiae* in BioGRID [52]. For genes lacking functional annotations, GO-term enrichment among predicted interaction partners was tested gene-by-gene with Fisher’s exact test and Bonferroni correction; significance was set at adjusted *P* < 1 × 10⁻¹⁰. Additional annotations were supplemented with MapMan Mercator4 v7.0 [53]. For genes still lacking functional assignments, additional predictions were obtained with PANNZER2 [54], the OMA browser [55] functional annotation tool, and STRING v12.0 [56]. The clusterProfiler R tool was used for GO enrichment analysis and visualization [57]. Genes with an FDR < 0.05 were considered significantly enriched in the target gene set compared to the background gene set. The enrichment score was calculated based on the ratio of genes in the target gene set divided by the ratio of genes in the background gene set.

### Protein ortholog finding using reciprocal best BLAST hit

To compare spliceosomal components between species, data on spliceosomal proteins in *Homo sapiens* was downloaded from the Spliceosome Database as a reference [58]. The protein fasta files of other model species (*Mus musculus, Danio rerio, Drosophila melanogaster, Caenorhabditis elegans, Saccharomyces cerevisiae, Arabidopsis thaliana*) were downloaded from the ENSEMBL database [59]. *Paramecium tetraurelia* protein fasta files were downloaded from ParameciumDB [60], and the protein fasta file of *Tetrahymena thermophila* was obtained from TGD Wiki [61]. Homologs of human spliceosomal proteins were then identified by performing reciprocal BLAST against the protein fasta files of each species [62]. To improve specificity and sensitivity, reciprocal best BLAST hits (RBBHs) were allocated using both blastp and mmseq easy-rbh.

After using *H. sapiens* as the reference species, the RBBHs of splicing-related proteins identified in the ciliate species *P. bursaria, P. tetraurelia*, and *T. thermophila* were then used as additional references to search for reciprocal best BLAST hits in the other ciliates using the same procedure. Non-synonymous substitution rates (Ka) were calculated for the ciliate species group (*P. bursaria, P. tetraurelia, T. thermophila, Ichthyophthirius multifiliis, Oxytricha trifallax, Stylonychia lemnae*), focusing on splicing-related genes that fall within the 26 core classes or families (Fig. 2B). Protein FASTA files and CDS FASTA files for *I. multifiliis, O. trifallax, and S. lemnae* were obtained from IchDB, OxyDB, and StyloDB, respectively [63–65]. Splicing-related proteins in these species were identified using blastp and mmseqs easy rbh against *H. sapiens* as the reference. PAL2NAL 14.1 was used to generate codon alignments for orthologs in the ciliate group, and PAML CODEML 4.10.9 was used to calculate pairwise Ka values between orthologous species [66–68].

### snRNA discovery in *P. bursaria*

The UsnRNA multiple sequence alignment in Stockholm format was retrieved from Rfam database 14.10 [69]. The INFERNAL program v1.1.5 was used to create a covariance model, to calibrate the model, and to search the *P. bursaria* genome for UsnRNA sequences employing the cmbuild, cmcalibrate, and cmsearch features, respectively [70]. Significant hits against UsnRNA sequences discovered by INFERNAL were extracted using BEDTools intersect [71]. The sequence of each UsnRNA candidate was then sought in R2DT software, together with the corresponding template, to obtain secondary structures in DBN format [72]. The structure was then annotated in RNAcanvas [73].

### Linear regression analysis of the relationship between PSI and splicing-related gene expression in *P. bursaria*

To elucidate the potential regulatory effect of splicing-related DEGs on identified DSIs, we performed a linear model:


\begin{eqnarray*}
{\mathrm{PSI}} &=& {{{\mathrm{\beta }}}_1} \cdot {\mathrm{\ }}\ln \left( {{\mathrm{SF\ normalized\ counts}} + 1} \right)\\&&+ {{{\mathrm{\beta }}}_2} \cdot \left( {{\mathrm{cell\ type}}} \right) + {{{\mathrm{\beta }}}_0} + \ \varepsilon,
\end{eqnarray*}

in which PSI values and ln(SF normalized counts + 1) were standardized using z-transformation across 48 replicates from 16 samples. The significance of the regression coefficients ${{\beta }_1}\ $and ${{\beta }_2}$ were assessed with FDR correction at a threshold of <0.1. Since DSI values and splicing-related DEGs were identified from comparisons between different endosymbiotic states (symbiotic and aposymbiotic cells), we accounted for a potential cell type confounding effect by setting ${{\beta }_1}$ to 0 when ${{\beta }_2}$ was found to be significant in the linear model. We interpreted a significant non-zero outcome for ${{\beta }_1}$ as being indicative of a potential regulatory effect of splicing-related gene expression on differential splicing.

### Intron orthologs between ciliate species and intron age assignment

The process of identifying intron orthologs was adapted from a method described previously [74]. Initially, protein homologs were retrieved using MMseqs2 easy-rbh to obtain reciprocal best-hit (RBH) proteins between species [75]. Next, the amino acid sequences of exon-exon junctions, comprising the 10 amino acids on either side of the junction, were extracted to define intron positions. These intron position sequences from protein homologs were then subjected to pairwise alignment using the pairwiseAlignment function in the Biostrings R package, using the BLOSUM-62 scoring system. Intron alignments were considered RBHs if their aligned regions exhibited at least 40% sequence identity and achieved the highest alignment score in both forward and reverse directions.

For introns found in orthologous genes shared at least between three out of four examined species, we determined their evolutionary age using phylostratigraphy and the maximum parsimony method [76, 77]. For this analysis, the youngest intron group, assigned an age of 1, is specific to the reference species, whereas the oldest intron group consists of introns originating from the first node in the phylogenetic tree examined.

## Results

### The extremely short introns in *P. bursaria* enhance gene expression

**Figure 1.**
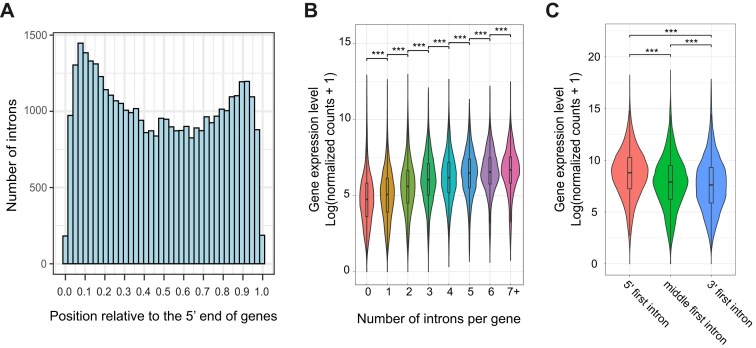
Intron position and intron-mediated enhancement of gene expression in *P. bursaria*. (**A**) The distribution of introns is enriched at both the 5′ and 3′ ends of genes. Distribution of introns relative to the 5′ end of protein-coding regions. Intron positions were determined by dividing the distance from the intron to the first codon by the total gene length. (**B**) Gene expression levels are positively correlated with the number of introns per gene. (**C**) Genes having the first intron at their 5′ end display significantly higher expression than the other two groups. Genes were grouped according to the position of their first intron. ***, *P*-value < .001, Mann–Whitney U test.

To characterize the features of *P. bursaria* introns, we annotated 39,715 introns in the functional gene set from the DK2 genome annotation file (Supplementary Table S1; see the “Materials and Methods” section for details). More than 80% of *P. bursaria* genes contain at least one intron, with an average of 2.7 introns per gene (Supplementary Fig. S1A). Moreover, we discovered a unique distribution of introns in *P. bursaria* transcripts, being enriched at both the 5′ and 3′ gene ends (Fig. 1A). A bias of introns toward the 5′ end of transcripts has also been reported for other model species, such as the budding yeast, fruit fly, and mouse [78], implying a general feature of eukaryotic introns. A majority of *P. bursaria* introns are < 40 nt in length, with a median length of 24 nt, and minimum and maximum lengths of 15 and 100 nt, respectively (Supplementary Fig. S1B). The distribution of intron lengths is very similar to that of *Paramecium tetraurelia*, which displays a median intron length of 25 nt [60]. In Supplementary Fig. S1C, we present a sequence logo plot showing that most of the annotated introns in *P. bursaria* are canonical introns with conserved GT-AG 5′ and 3′ splice boundaries, indicating that an exclusively U2-dependent splicing mechanism operates. While the GC content of *P. bursaria* exons is ∼30.6%, the GC content of its introns is substantially lower (average = 17.7%) (Supplementary Fig. S1D).

To compare how splicing is physiologically regulated between symbiotic and aposymbiotic cells, we performed transcriptomics analysis on samples collected from both cell types at eight time points (8 AM, 11 AM, 2 PM, 5 PM, 8 PM, 11 PM, 2 AM, and 5 AM) through a light-dark cycle. *Paramecium bursaria* exhibits robust circadian behaviors that are entrained by light–dark cues [79, 80]. Importantly, we did not apply external cell-cycle synchronization, as such treatments would disrupt the tightly coupled host–symbiont division dynamics and potentially confound the physiological regulation we sought to measure [81–83]. We used the resulting data to explore the characteristics of small intron splicing in *P. bursaria*. In eukaryotes, intron splicing plays a crucial role in regulating gene expression through the co-transcriptional splicing mechanism [84]. We investigated if the small introns in *P. bursaria* exert a similar function. Transcriptomic data from all 16 samples were pooled together and analyzed. We observed that genes containing introns exhibited significantly higher expression levels than those without introns (Wilcoxon rank-sum test, *P* < .001). Moreover, gene expression level was positively correlated with intron number (Fig. 1B).

This positive correlation between intron numbers and gene expression levels in *P. bursaria* implies intron-mediated gene expression enhancement. U1 snRNPs can directly recruit transcription factors, such as TFIIH, TFIIB, and TFIID, to a gene promoter when the intron 5′ splice site is located near the promoter [85, 86]. Since we detected a bias in intron positions toward the 5′ end of transcripts (Fig. 1A), we investigated if the presence of introns at this position is associated with enhanced gene expression in *P. bursaria*. To do so, we grouped and compared *P. bursaria* genes based on the relative position of the first intron: the 5′ end group (i.e. the first intron is located within 0%–25% of the total length), the middle group (within 25%–75% of total length), or the 3′ end group (within 75%–100% of total length). Indeed, the 5′ end group displayed significantly higher expression than the other two groups (Fig. 1C).

### Conservation of the U2-dependent spliceosome in *P. bursaria*

**Figure 2.**
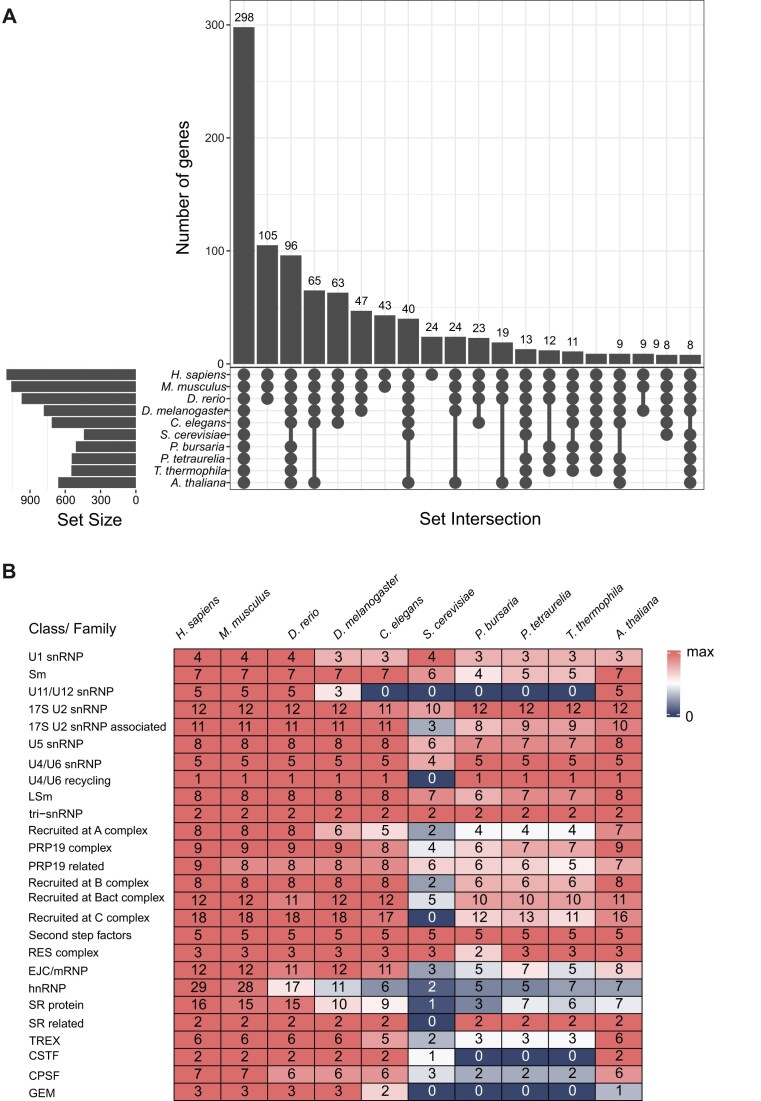
Conserved splicing-related proteins in model organisms and ciliate species. (**A**) An UpSet plot displaying the numbers of splicing-related proteins shared among 10 species. Set size represents the number of splicing-related proteins in each species, with numbers above the bars indicating the count of proteins in each shared group. (**B**) Number of splicing-related proteins in each protein class/family across 10 species. Colors indicate protein counts relative to the median: red (above median), white (equal to median), and blue (below median).

Compared to other eukaryotes having well-characterized splicing mechanisms [1, 87], intron size in *P. bursaria* is relatively small (Supplementary Fig. S1B). Spliceosomes are huge protein complexes containing many snRNAs and proteins. The human spliceosome A complex is ∼205 × 195 × 150 Å in size and covers 79–125 nt of a stretched RNA, based on its cryogenic electron microscopy structure [88]. The *P. bursaria* spliceosome likely requires extensive reorganization to function on small introns. To investigate this possibility, we downloaded 1,005 human splicing-related proteins from SpliceosomeDB [58]. We identified orthologs across three representative ciliate species (*P. bursaria, P. tetraurelia*, and *T. thermophila*), along with other model organisms, including *M. musculus, D. rerio, D. melanogaster, C. elegans, S. cerevisiae*, and *A. thaliana* (Supplementary Table S2) [75]. Among all of the species we analyzed, 298 out of 1,005 spliceosomal proteins were shared, representing core spliceosome components (Fig. 2A).

Forty human splicing-related proteins were not identified in the ciliate species, despite being present in all other examined eukaryotes, including components of U1 snRNP (e.g. LUC7L), U5 snRNP (e.g. CD2BP2), and LSm protein (e.g. LSM1) (Fig. 2A and Supplementary Table S3). The lack of detectable homologs may reflect human-specific gene gains, lineage-specific gene loss in ciliates, or sequence divergence beyond the sensitivity of our homology search pipeline (see the “Materials and methods” section). In human cells, LUC7L enhances splicing by recognizing a strong consensus sequence downstream of the 5′ splice site (5′SS, within the intron), whereas its paralog LUC7L3 promotes splicing by interacting with an upstream consensus sequence located within the exon [89, 90]. Loss of LUC7L and retention of LUC7L3 in *P. bursaria* corresponds to it possessing a conserved upstream consensus (positions −2 and −1 in the exon) and lacking a downstream consensus (positions +3 to +6 in the intron) to the 5′ splice site (Supplementary Fig. S1C), implying a streamlined mechanism of 5′SS recognition by the U1 snRNP, likely a consequence of the reduced splicing machinery possessed by ciliates.

Interestingly, several factors known to facilitate short-intron splicing in human cells are conserved in ciliates. In particular, SMU1, which bridges U2 and the tri-snRNP on introns with a short 5′SS-to-BPS distance [91], and RBM17, which replaces U2AF2 to stabilize U2 on short polypyrimidine tracts near the 3′SS [92], are both present. Their conservation suggests that mechanisms that stabilize the spliceosome on short introns may be shared between ciliates and humans. In contrast, we observed a complete absence of the U11/U12 snRNP, CSTF, and GEM complexes among the ciliates we considered (Fig. 2B). The lack of U11/U12 corresponds to the absence of minor (AT–AC) introns in ciliates. The GEM complex, which facilitates maturation of snRNPs [93, 94], is also missing. In *Arabidopsis* and human cells, partial loss or overexpression of GEM has been associated with increased intron retention [95, 96]. An alternative, GEM-independent snRNP assembly pathway has been proposed in *S. cerevisiae* [97]; however, whether a similar mechanism exists in ciliates remains unclear.

Additionally, we uncovered a reduction in the number of proteins associated with regulatory classes, including SR proteins, hnRNPs, and CPSF (Fig. 2B). SR proteins and hnRNPs play key roles in exon–intron definition [98], and their partial absence suggests that the low-GC ciliate genomes may rely less on complex SR/hnRNP networks to demarcate exon boundaries. CSTF and CPSF, which mediate 3′-end cleavage during pre-mRNA maturation [99, 100], are also missing, implying that ciliates may have evolved a distinct mRNA 3′-end processing mechanism.

Notably, although the numbers of splicing-related proteins in ciliates and *S. cerevisiae* are relatively similar, yeast and ciliate species possess different sets of unique proteins (Fig. 2A and Supplementary Table S3), likely reflecting different trajectories of spliceosome reorganization, one pertaining to the reduction in intron number displayed by *S. cerevisiae* and another for the reduction in intron size of ciliates. In addition, we identified a small subset of genes with inconsistent conservation across ciliate species; non-synonymous substitution rate (Ka) analyses indicate that these genes are evolving more rapidly (Supplementary Fig. S1E).

Overall, the numbers of U2-dependent core spliceosome proteins in *P. bursaria*, including U1, U2, U4, U5, U6, Sm, and Lsm proteins, are conserved. To further investigate the snRNA repertoire in *P. bursaria*, we analyzed conserved secondary structures of UsnRNAs from the Rfam database [69]. We detected U1, U2, U4, U5, and U6 snRNAs with conserved secondary structures and key functional motifs, but not the U11 or U12 snRNAs (Supplementary Fig. S2). Together, our data indicate that a modified U2-dependent splicing mechanism operates in *P. bursaria* to splice small introns.

### Intron retention and alternative 3′ splice sites are the most abundant alternative splicing events in *P. bursaria*

**Figure 3.**
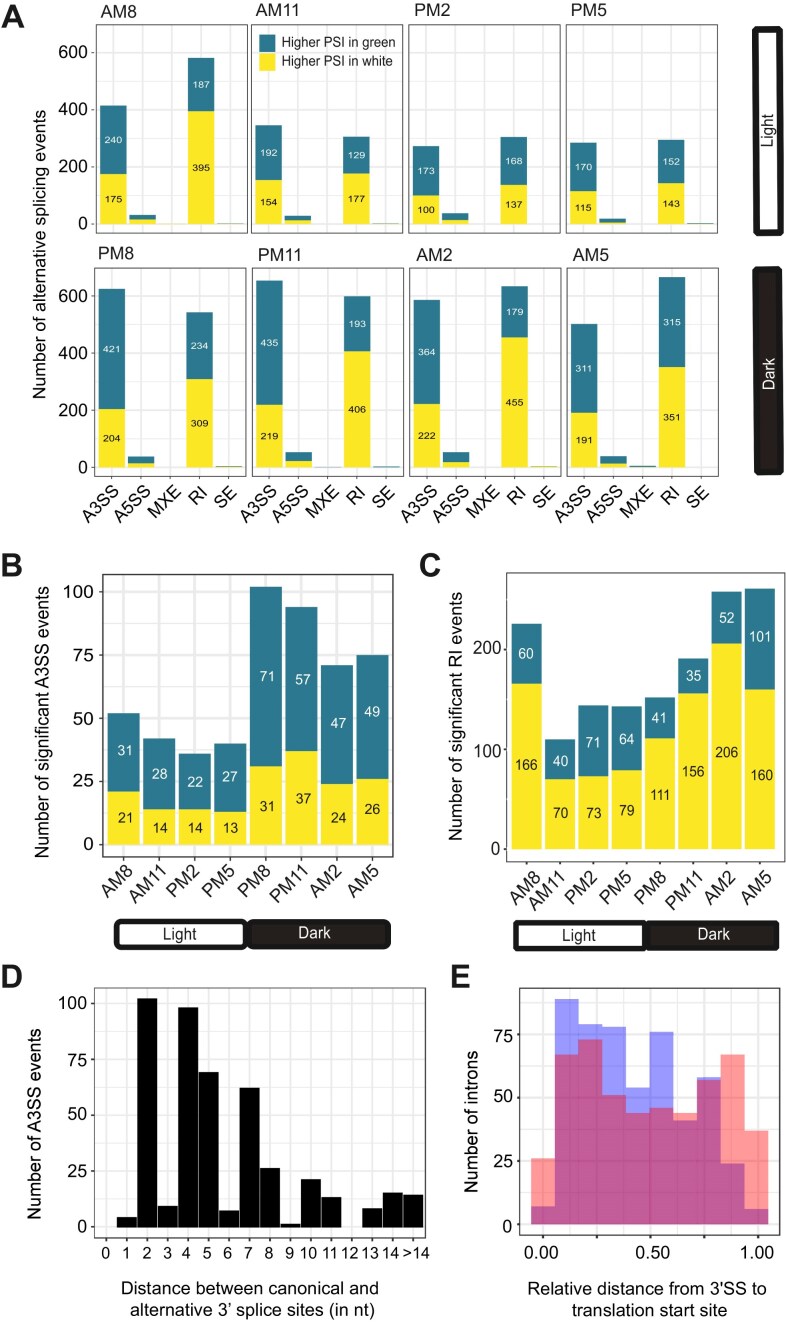
Alternative splicing events between symbiotic and aposymbiotic *P. bursaria* cells. (**A**) Intron retention and alternative 3′ splice site events are the most frequently regulated between symbiotic and aposymbiotic cells. Numbers of significant alternative splicing events (FDR < 0.05) between symbiotic and aposymbiotic cells at eight time points through a single 24-h light-dark cycle. Note that some introns were alternatively spliced at multiple time points. Teal blue indicates higher PSI in symbiotic cells and yellow indicates higher PSI in aposymbiotic cells. The alternative splicing events are A3SS: alternative 3′ splice site, A5SS: alternative 5′ splice site, MXE: mutually exclusive exon, RI: intron retention, and SE: exon skipping. (**B**) Number of significant alternative 3′ splice site (A3SS) events between symbiotic and aposymbiotic cells. (**C**) Number of DSIs between symbiotic and aposymbiotic cells. (**D**) Distance (in nucleotides) between the alternative 3′ splice site and the canonical 3′ splice site for significant A3SS events between symbiotic and aposymbiotic cells. (**E**) Introns displaying A3SSs are significantly enriched in the 5′ portion of genes (Kolmogorov–Smirnov test, *P*-value = 7.599e^−07^). Blue indicates introns with an A3SS and orange represents the same number of introns randomly selected from among all introns.

Since alternative splicing influences both protein isoform diversity and gene expression levels, it may play a crucial role in adaptive regulation of the endosymbiotic process. To explore this possibility, we analyzed how five major types of alternative splicing events are regulated across eight time points between symbiotic and aposymbiotic *P. bursaria* cells: intron retention (RI), exon skipping (SE), alternative 3′ splice site (A3SS), alternative 5′ splice site (A5SS), and mutually exclusive exon (MXE) (Supplementary Fig. S3). Our results show that intron retention and alternative 3′ splice site events were the most frequently regulated between symbiotic and aposymbiotic cells (Fig. 3A). We then filtered for significant A3SS and RI events (FDR < 0.05) using junction read counts (total junction reads ≥ 10) and PSI changes (|∆PSI| ≥ 0.1), which uncovered 512 significant A3SS events (Fig. 3B) and 992 significant RI events (Fig. 3C).

Interestingly, significant A3SS and RI events proved to be more frequent at night (8 PM, 11 PM, 2 AM, 5 AM) than during the day (8 AM, 11 AM, 2 PM, 5 PM) (Fig. 3B and C). Moreover, during night-time points, a higher ratio of A3SS events presented increased PSI in symbiotic cells relative to aposymbiotic cells (Fig. 3B). In contrast, RI events exhibited a higher proportion of increased PSI in aposymbiotic cells at night (Fig. 3C). Since *P. bursaria* cells exhibit rhythmic physiological changes during the day-night cycle [101], our results indicate that alternative splicing may be involved in these changes.

To assess the functional impact of the alternative splicing events, we investigated the impact of A3SS events on the translation reading frame by calculating the distance between canonical 3′SS and alternative 3′SS. Unexpectedly, we found that the majority of distances were not multiples of nucleotide triplets (*n* = 479 out of 512 events), which would cause a frameshift in open reading frames and potentially trigger mRNA degradation via the NMD pathway (Fig. 3D). This outcome differs from the 3′ wobble splicing observed in mammalian cells (e.g. NAGNAG motifs), which often preserves functional reading frames from both 3′SS choices [102, 103]. Furthermore, we observed that A3SS events occur more often in the introns located in the 5′ portion of genes (Fig. 3E). In addition, exon junction presence is significantly more common downstream of A3SS event introns compared to random introns (odds ratio = 1.97, Fisher’s exact test *P*-value = 1.101e^−06^). Taken together, these results indicate that A3SS selection in *P. bursaria* predominantly serves as a mechanism to abrogate protein production.

### Intron retention rates are associated with GC content, intron length, and gene expression levels

Next, we investigated the intrinsic factors influencing intron retention rate in *P. bursaria*. After filtering out the introns with low junction read counts (i.e. read count ≤ 10), 66% of total introns (*n* = 26,123 out of 39,715) presented sufficient counts to calculate splicing efficiency in at least one sample. More than 87% of these introns exhibited intron retention rates (presented as PSI) lower than 0.1 for all samples, indicating that most introns in *P. bursaria* are spliced efficiently (Supplementary Fig. S4A and Table 4).

**Figure 4.**
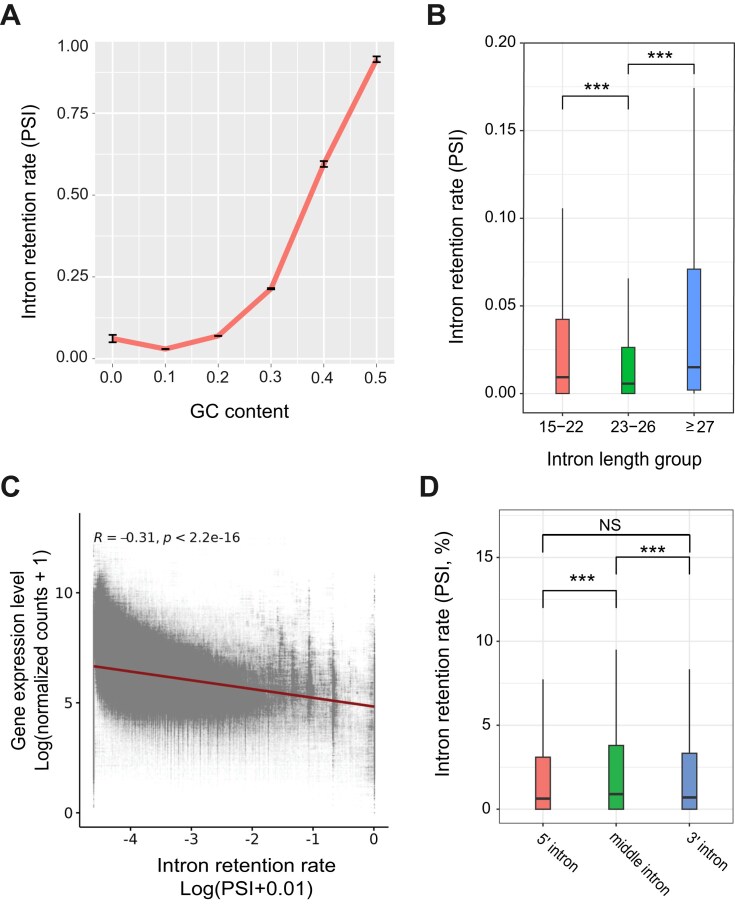
The intron retention rate correlates with intron sequence features and gene expression. (**A**) The intron retention rate is positively correlated with the GC content of introns. GC content values have been rounded to the nearest group. Error bars indicate standard error of the mean. (**B**) Intron retention rates across different intron length groups. The introns were divided into three groups, 15–22 nt, 23–26 nt, and ≥27 nt, representing approximately the first, middle two, and last quartiles of all introns. ***, *P*-value < .001, Wilcoxon rank-sum test. (**C**) Gene expression levels are negatively correlated with intron retention rates, based on a Pearson correlation analysis. (**D**) 5′ end and 3′ end introns are spliced more efficiently than middle introns. Introns were classified as 3′ introns (relative position ≥ 0.75), 5′ introns (relative position ≤ 0.25), or middle introns (0.25 < relative position < 0.75). ***, *P*-value < .001; NS, *P*-value > .05, Mann–Whitney U test.

We detected a strong positive correlation between the GC content of introns and intron retention rates (Fig. 4A). Although a similar trend has been reported previously for *P. tetraurelia* [39], the effect of GC content on intron retention appears to be much stronger in *P. bursaria*. In addition to intron GC content, we found that intron length also contributes to splicing efficiency. Introns larger than 26 nt or smaller than 23 nt were spliced less efficiently than introns of 23–26 nt, representing the middle 50% of all introns (Fig. 4B).

As shown in Fig. 1, genes with a higher intron count and the first intron positioned near the 5′ end of genes tend to display elevated expression levels. In addition, we observed a negative correlation between expression level and the intron retention rate (Fig. 4C), indicating that efficient splicing contributes to transcript abundance. Moreover, introns at the 5′ and 3′ ends are spliced more efficiently than introns in the middle of genes (Fig. 4D), consistent with their positional enrichment and potential roles in regulating transcript maturation (Fig. 1A and C). These results highlight that intron splicing, especially for introns located at the 5′ end of genes, is associated with enhanced gene expression in *P. bursaria*, as also reported previously for mammalian cells [62, 104].

### Symbiotic and aposymbiotic cells display two distinct intron splicing patterns associated with differential expression of splicing factors

**Figure 5.**
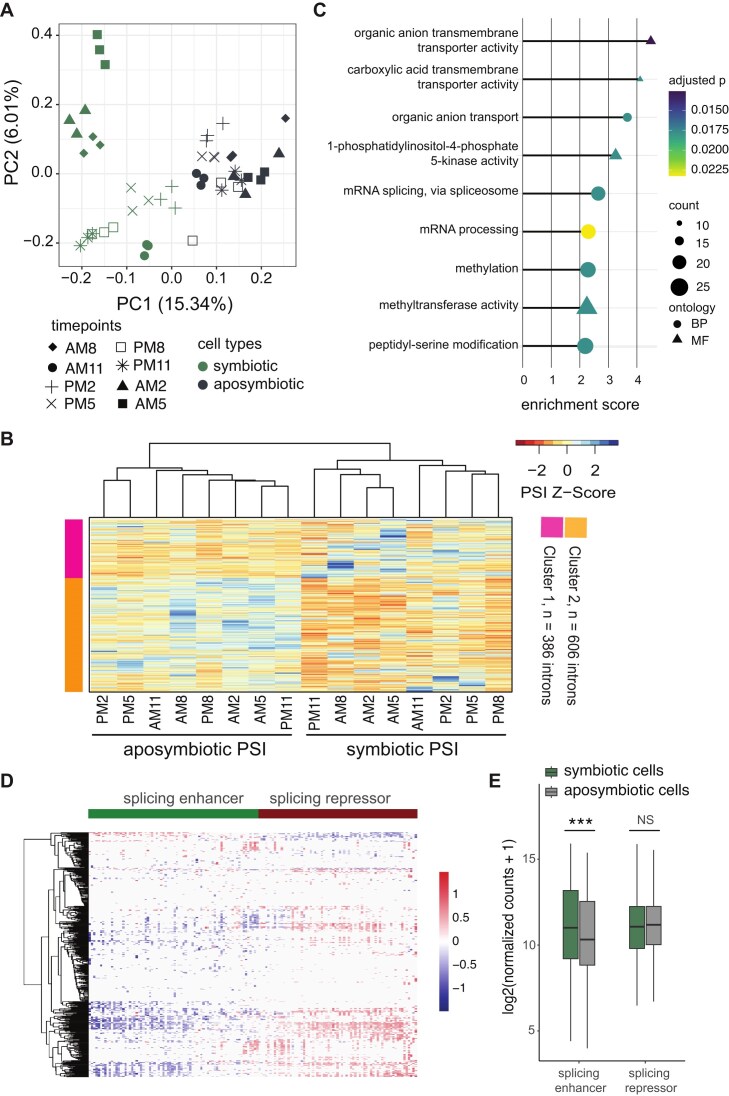
Patterns of intron splicing between symbiotic and aposymbiotic cells. (**A**) Symbiotic and aposymbiotic samples are clearly separated by PCA on the intron retention rates of 992 DSIs. Each time point sample comprised three biological replicates. (**B**) k-means clustering separates the identified DSIs into two clusters displaying distinct patterns of intron retention (PSI). The heatmap shows the PSI values for 16 samples in two DSI clusters. Color is scaled according to PSI *z*-score. Cluster 1 (pink), *n* = 386 introns; cluster 2 (orange), *n* = 606 introns. (**C**) GO term enrichment for genes containing DSIs. BP indicates biological process, and MF indicates molecular function. (**D**) Association of DSIs with the expression levels of splicing-related genes. Heatmap shows the coefficients (${{{\mathrm{\beta }}}_1})$ of splicing-related genes from the linear model of DSI PSI values, based on 992 DSIs (rows) and 147 differentially expressed splicing-related genes (columns). These splicing-related genes can be generally classified into two distinct groups, i.e. splicing enhancers and repressors. (**E**) Splicing-enhancing factors are significantly upregulated in symbiotic cells. Gene expression levels of splicing enhancers and repressors in symbiotic and aposymbiotic cells are shown. ***, *P*-value < .001; NS, *P*-value > .05, Wilcoxon rank-sum test.

To investigate the intron splicing pattern further, we compiled all DSIs (|∆PSI| ≥ 0.1 and FDR < 0.05) from all time points (Fig. 3C), resulting in a total of 992 DSIs in 883 genes (Supplementary Table S5). A principal component analysis (PCA) was then performed to cluster the samples (16 samples, each with three replicates) based on the PSI values of these DSIs. The PSI profiles of the DSIs effectively distinguished symbiotic from aposymbiotic cells (Fig. 5A), indicating that endosymbiosis influences the splicing pattern. Additionally, symbiotic cells separated into two sub-clusters along PC2 (Fig. 5A). One sub-cluster comprised 2 AM and 5 AM (nighttime) and 8 AM (early daytime), while the other included 11 AM, 2 PM, 5 PM, 8 PM, and 11 PM (early nighttime). This pattern may reflect a delayed RNA splicing response to light transitions, potentially associated with photosynthetic activity of the endosymbiotic algae.

Then, we performed k-means clustering to separate the identified DSIs into two clusters having distinct intron PSI patterns (Fig. 5B and Supplementary Table S5). Cluster 1 consists of DSIs with higher PSI in symbiotic cells (*n* = 386 introns), whereas cluster 2 (*n* = 606 introns) includes DSIs with higher PSI in aposymbiotic cells (Fig. 5B). GO term enrichment analysis of genes containing DSIs revealed an overrepresentation of mRNA splicing and transport-related terms, including organic anion transmembrane transporter activity, organic anion transport, and carboxylic acid transmembrane transport (Fig. 5C and Supplementary Table S6). Notably, DSIs positively correlated with gene expression are enriched with frame-preserving events (Fisher’s exact test, *P* = .03), suggesting that a subset of these in-frame introns may be translated into functional peptides (Supplementary Fig. S4B). Furthermore, these DSIs are enriched in splicing-related pathways, implying that the splicing program represents one of the earliest functional groups shaped by alternative splicing (Supplementary Fig. S4B and Supplementary Table S7).

Previous studies have shown that endosymbiotic algae are maintained in a membranous compartment (i.e. the perialgal vacuole) within *P. bursaria* host cells, and that the host and endosymbionts continuously exchange several organic compounds to establish a stable mutualistic relationship [26]. We further identified 38 DSIs in transport-related genes that, although differentially used between symbiotic and aposymbiotic cells, preserved the reading frame and did not alter gene expression levels (Supplementary Table S8). These introns are likely retained to generate alternative protein isoforms with distinct regulatory or functional properties. Together, our findings indicate that intron splicing may play a pivotal role in regulating these transmembrane exchanges. Moreover, since intron retention rates in spliceosome genes appear to be regulated, there is potentially autoregulation of splicing-related genes in the context of endosymbiosis, which may in turn influence the retention levels of other introns.

Certain splicing factors can regulate the introns of genes involved in specific cellular pathways. For instance, HNRNPK and SRSF1 were shown previously to control intron retention during B cell development [105]. Since we found that intron splicing efficiency is regulated in endosymbiosis, potential splicing factors may influence splicing of DSIs during this process. To explore this supposition, we analyzed 6949 DEGs between symbiotic and aposymbiotic cells across all time points and identified 147 splicing-related genes (Supplementary Table S9; see the “Materials and methods” section for details). Next, we used a linear regression model to elucidate the potential regulatory effect of the 147 splicing-related DEGs on 992 DSIs.

Since we identified both differentially expressed splicing factors and DSIs in our comparisons of symbiotic and aposymbiotic cells, the status of endosymbiosis may confound any associations. To account for this issue, we incorporated endosymbiotic status as a covariate in the model (DSI ∼ splicing-related DEG + endosymbiotic status) and retained only DSIs whose coefficients were significant for splicing-related DEGs but not for endosymbiotic status (Supplementary Table S10; see the “Materials and methods” section for details). Applying this filter yielded 477 introns, which likely represent direct regulatory targets of these splicing factors (Fig. 5D). We could classify the 147 splicing-related genes into two groups based on their mean correlation coefficient with intron retention rates (PSIs): splicing-enhancing factors, whose mean coefficient with the PSI of DSIs is negative, and splicing-repressing factors, whose mean coefficient is positive with PSI (Fig. 5D). Among the enhancers, SNRNP70 and SNRPC are core components of the U1 snRNP that facilitate 5′SS recognition. SNRPC directly interacts with the 5′SS-U1 snRNA duplex to stabilize the early spliceosome [106]. In addition, SNRPD1 is a constituent of the Sm protein ring that encircles U1, U2, U4, and U5 snRNAs and is essential for spliceosome assembly [107]. Together, these factors act coordinately to promote efficient splicing (Supplementary Fig. S4C). Notably, splicing-enhancing factors were significantly upregulated in symbiotic cells (Fig. 5E), implying that symbiotic cells regulate these splicing-related factors to fine-tune the splicing and expression of intron-containing genes.

### Intron evolution in ciliate species reveals higher splicing efficiency and lower intron GC content in aged introns

We observed that some intron characteristics, such as GC content and the intron distribution within genes, are closely associated with splicing efficiency and gene expression regulation (Figs 1 and 3). Moreover, symbiotic cells may adjust the expression of specific splicing factors to regulate splicing efficiency and mRNA levels (Fig. 5). Given the functional significance of introns, we explored the evolutionary patterns of introns within the genus *Paramecium*.

For this analysis, we selected three Paramecium species with well-annotated genomes, i.e. *P. bursaria, P. tetraurelia*, and *P. caudatum*, with *T. thermophila* acting as an outgroup. We identified 8,886 orthologs shared across these species. We then identified the best-matching introns from paired orthologous genes between *P. bursaria* and the other species (see the “Materials and methods” section for details).

**Figure 6.**
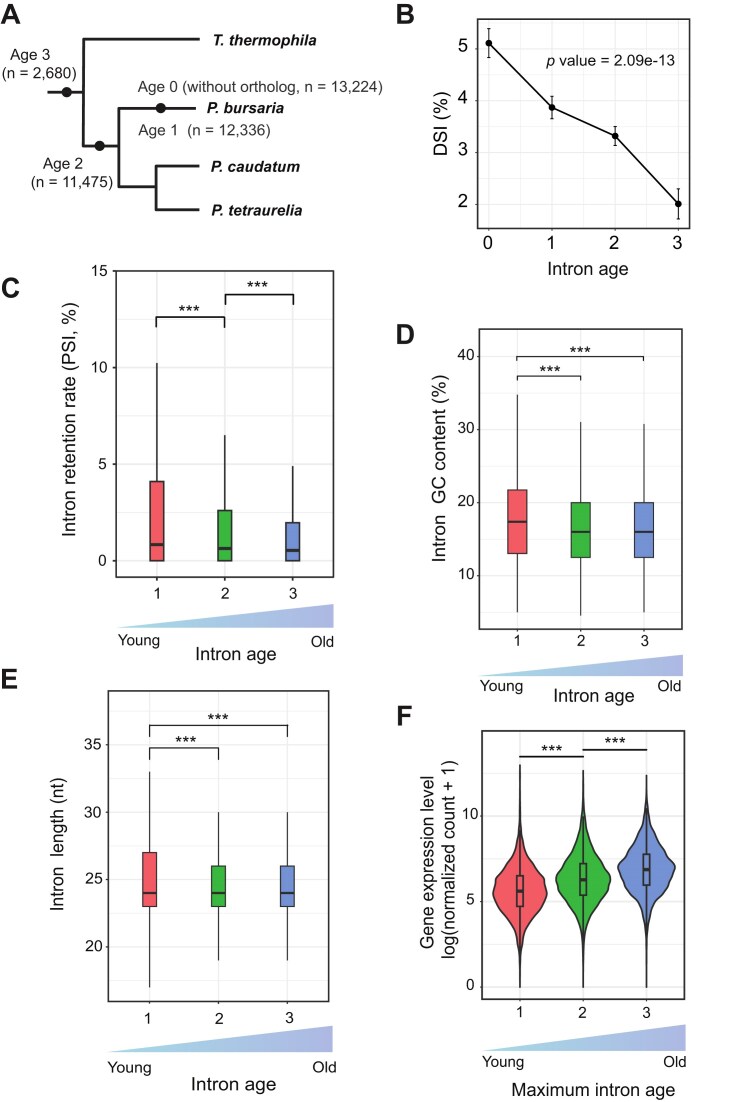
Short intron evolution in *P. bursaria*. (**A**) A diagram showing the phylogenetic relationships between different *Paramecium* and *Tetrahymena* species. Introns in *P. bursaria* were assigned to different age groups based on their conservation between *P. bursaria* and the other species. The Age 0 group represents the youngest introns in the genes without orthologs, whereas the Age 3 group represents the oldest introns. *n* indicates the intron number in each group. (**B**) Percentage of DSIs in each intron age group. *P*-value was determined by the Chi-square trend test. Error bars represent the standard error, calculated as √(p(1–p)/n), where p is the proportion of DSIs and n is the total number of introns in each age group. (**C**) Young introns exhibit greater intron retention than old introns. Boxplots show the intron retention rate for each intron age group. ***, *P*-value < .001, Mann–Whitney U test. (**D**) Young introns display a higher GC content than old introns. Boxplots show the intron GC content for each intron age group. ***, *P*-value < .001, Mann–Whitney U test. (**E**) Young introns present a wider intron size distribution than old introns. Boxplots show intron length (in base pairs) for each intron age group. ***, *P*-value < .001, Kolmogorov–Smirnov test. (**F**) Genes containing old introns are more highly expressed than those solely possessing young introns. Boxplots show expression level for each gene group based on the maximum intron age in the genes. ***, *P*-value < .001, Mann–Whitney U test.

To classify intron origins, we applied phylostratigraphy and maximum parsimony, assigning introns to different nodes on the phylogenetic tree. We identified 13,224 introns located within genes lacking detectable orthologs, which we designated as age group 0. Among introns residing in orthologous genes, 46.6% are unique to *P. bursaria* (*n* = 12,336, age group = 1), 43.3% are specific to the *Paramecium* lineage (*n* = 11,475, age group = 2), and only 10.1% belong to the oldest group (*n* = 2,680, age group = 3), highlighting dynamic evolution of introns in this lineage (Fig. 6A).

Interestingly, DSIs are significantly depleted among older intron groups (Fig. 6B). Age 3 DSIs are enriched in genes associated with spliceosome and mitochondrial functions, whereas younger DSIs are predominantly involved in transporter activity and nutrient metabolism (Supplementary Table S11), suggesting context-dependent coevolution of DSIs to facilitate metabolic adaptation during endosymbiosis. Consistently, older introns (age groups 2 and 3) exhibit better splicing efficiency than newer introns (age groups 0 and 1) (Fig. 6B). In *P. bursaria*, high-GC introns generally have higher PSI values compared to low-GC introns. This scenario aligns with our observation that new introns in *P. bursaria* have higher GC contents than older ones (Fig. 6C). We also observed a trend whereby younger introns present a wider range of length distributions compared to introns in the older groups (Fig. 6D). Consistently, we detected that introns longer than 27 nt or shorter than 23 nt are spliced less efficiently compared to those within the 23–26 nt range (Fig. 4B), indicating that the intron length distribution in *P. bursaria* has evolved over time toward an optimal range. Given the positive association between intron presence, intron number, and gene expression, we assessed gene expression in relation to intron age, reflecting when introns were acquired in the evolutionary tree. We categorized the 8,886 orthologous genes into three groups based on the age of their oldest intron: group 1 comprised genes with only young introns (*n* = 1,466); group 2 included genes with a maximum intron age of 2 (*n* = 4,863); and genes with a maximum intron age of 3 were assigned to group 3 (*n* = 1,656). Notably, genes hosting older introns exhibited significantly higher gene expression levels compared to those with only young introns (Fig. 6E). This finding further supports a role for introns in enhancing gene expression.

To determine if the evolutionary patterns we observed in *Paramecium* introns are also present in other ciliates, we extended our analysis to *Tetrahymena*. We selected four representative species from genus *Tetrahymena*, including *T. thermophila, T. malaccensis, T. eliotti*, and *T. borealis*. Using the same approach as performed for *Paramecium*, we classified introns in *T. thermophila* that had gene orthologs in at least three *Tetrahymena* species into four intron age groups (Supplementary Fig. S5A).

Our results revealed that the majority (68.9%) of introns in *T. thermophila* belonged to the oldest group (*n* = 42,030 of a total of 60,971 introns). In contrast, species-specific introns (age group 1) in *T. thermophila* made up only 9.3% of the total introns in shared orthologous genes (*n* = 5,678 introns) (Supplementary Fig. S5A). We found that younger introns also exhibited a higher retention rate, a pattern consistent with our result for *P. bursaria* introns (Supplementary Fig. S5B). Similarly, young introns in *T. thermophila* had a higher GC content and a wider length range compared to older introns (Supplementary Fig. S5C and D). When we categorized genes based on their oldest intron age in *T. thermophila*, we observed a significant trend whereby genes containing older introns exhibited higher expression levels compared to those with only younger introns (Supplementary Fig. S5E). Taken together, our analysis of intron features across different evolutionary age groups reveals a consistent pattern of intron optimization, characterized by improved splicing efficiency and enhanced gene expression, during evolution of both the *Paramecium* and *Tetrahymena* lineages.

## Discussion

### Alternative splicing as a mode for gene regulation in endosymbiosis

Host cells integrating symbionts likely experience drastic changes in gene expression. A previous study on *P. bursaria* uncovered nearly 7,000 DEGs between symbiotic and aposymbiotic cells [30], which is consistent with our results presented herein. Another study indicated that upregulation of glutamine biosynthesis is correlated with symbiont abundance [27], supporting that gene regulation plays a crucial role in endosymbiosis. Similar phenomena have been observed in other examples of algal secondary endosymbiosis. For example, genes involved in glutamine synthesis and phosphate transporters are also upregulated in *Hydra viridissima* hosting algal endosymbionts [108]. In addition to transcriptional regulation, post-transcriptional regulation, such as alternative splicing, can provide the host with more flexibility in adjusting the endosymbiotic relationship, especially when the endosymbiotic algae undergo physiological changes relating to photosynthesis during the day/night cycle. A recent study on *P. bursaria* found that 6mA DNA methylation facilitates intron retention and may serve as a reverse marker to distinguish endosymbiosis-related genes [109], providing a potential link between splicing regulation and endosymbiosis.

In our study, we identified 992 introns that are differentially spliced and 512 A3SS events between symbiotic and aposymbiotic *P. bursaria* cells (Fig. 3). Among these, 94% of A3SS events (479 out of 512) introduce frameshifts, indicating that they likely regulate gene expression through RNA degradation. In contrast, intron retention events are not biased toward out-of-frame disruptions. Notably, several intron retention events within transport-related genes are in-frame without altering mRNA levels, suggesting that they may be translated to produce protein isoforms with distinct functional or regulatory properties. Together, our findings indicate that alternative splicing modulates both gene expression and protein function during endosymbiosis.

### Splicing factors specifically regulate DSIs between symbiotic and aposymbiotic cells

Alternative splicing is regulated by many factors, such as co-transcriptional, chromatin structure, DNA sequence, and epigenetic modifications, as well as splicing factors [110]. In *P. tetraurelia*, nucleosome positioning affects intron splicing, with introns at the edges of nucleosomes displaying greater splicing efficiency [39]. Moreover, 6mA DNA methylation in *P. bursaria* was shown previously to be enriched in retained introns [109]. Alternative splicing factors are regulated in a tissue-specific manner in humans. For example, expression of polypyrimidine tract binding protein 1 (PTB1) is high in neuron progenitor cells, but significantly downregulated in differentiated neurons [111, 112]. Since endosymbiosis induces many physiological changes in the host cell, including the formation of new organelles for endosymbionts, alternative splicing regulation during this process may be influenced by specific splicing factors. Our study has identified differentially expressed splicing-related genes between symbiotic and aposymbiotic cells, implying a potential role for these splicing factors in intron splicing during endosymbiosis. Furthermore, a linear regression model between splicing-related DEGs and DSIs across 16 samples revealed that a subset of the DSIs is strongly associated with those splicing-related DEGs. Two groups of splicing factors possibly represent “splicing enhancers” and “splicing repressors” of those DSIs in the endosymbiotic relationship (Fig. 5D).

To pinpoint key splicing factors strongly associated with the PSI of DSIs, we ranked 147 splicing factor DEGs based on the number of significant coefficients across introns (Fig. 5D and Supplementary Fig. S4B). The top 30 splicing-related genes include several ribosomal proteins and core splicing factors, such as U1 snRNPs (SNRNP70, SNRPC), an Sm protein (SNRPD1), a second-step spliceosome factor (PRPF18), RNA helicases (DDX23, DDX46), a U5 snRNP (TXNL4A), and chromatin-related proteins (SMARCA5, SMARCA1).

The expression of several ribosomal proteins is negatively associated with the PSI of potentially regulated introns, including RPL22, RPS20, and RPS11. Apart from their canonical function in protein translation, ribosomal proteins have also been shown to exert alternative splicing regulatory activity. For example, the large protein subunit L10a in nematodes and vertebrates can bind to its own pre-mRNA to switch splice site choice [113]. Although the exact regulatory functions of such ribosomal proteins remain to be studied further, it is possible that they act in alternative splicing regulation by binding to RNA or by interacting with other splicing factors.

For the top 30 genes associated with DSIs, we found that the coefficient relationships (i.e. either positively or negatively related to DSIs) of splicing factors aligned well with their known functions in splicing regulation. For instance, PRPF18 plays a crucial role in maintaining high splicing fidelity during the second splicing step, and G3BP2 interacts with PSF (polypyrimidine tract-binding protein-associated splicing factor) to enhance mRNA stability [114–116]. However, we also found that the coefficient relationship for some genes differed from their functions suggested by previous studies. For example, PRPF4B phosphorylates the SRSF1 protein, allowing it to work as a splicing enhancer in the fission yeast [117], yet we found that PRPF4B acts as a splicing repressor on DSIs in *P. bursaria*. Thus splicing regulation appears to operate differently in *P. bursaria*, particularly in the context of endosymbiosis. Further research is necessary to explore how the regulation of DSIs is involved in endosymbiosis.

### Intron evolution is closely linked to gene expression regulation

Although most introns are spliced efficiently in *P. bursaria*, we observed a significant difference between the splicing efficiency of old and newly acquired introns (Fig. 6). While not all individual differences are large in absolute magnitude, their cumulative effect contributes meaningfully to changes in gene expression, reflecting the complex regulatory landscape of splicing. We detected a similar pattern for *Tetrahymena* (Supplementary Fig. S5). This outcome indicates that introns gained over time can adapt to the splicing system, potentially through sequence modifications or changes in splicing factor binding or interactions [118, 119]. A previous study demonstrated that intron orthologs from various species lacking U2AF1 and having a shorter distance between the BPS and the 3′ splice site (3′SS) are spliced more efficiently in *S. cerevisiae* (which also lacks U2AF1 and has a short BPS-to-3′SS distance) compared to intron orthologs from species that possess U2AF1 orthologs and have a longer BPS-to-3′SS distance. This finding underscores specialization of the splicing machinery of a particular species for its own intron structures [118]. Our analyses consistently uncovered differences between new and old intron sequences, showing that new introns in *P. bursaria* have a higher GC content and a wider length distribution than old introns (Fig. 6). Intron GC content was shown previously to be negatively correlated with splicing efficiency in both *P. tetraurelia* and *S. cerevisiae* [39, 118]. We report herein a similar trend in *P. bursaria* (Fig. 4A). In terms of intron length, those of 23–26 nt in *P. bursaria* exhibit greater splicing efficiency than longer or shorter introns (Fig. 4B). A wider length distribution of new introns could also contribute to the reduced splicing efficiency we detected.

We also observed higher expression of genes containing old introns compared to genes only having new introns (Fig. 6E). Moreover, gene expression levels were positively correlated with intron number (Fig. 1B). Thus, it is clear that the presence of introns is tightly associated with gene expression levels. The reduced splicing efficiency of new introns may be attributable to new introns still undergoing optimization specific to the splicing machinery. Consequently, through further sequence modifications, they may ultimately behave like old introns. Alternatively, some new introns may have specific regulatory functions, providing the cell with a strategy to fine-tune splicing efficiency (and therefore gene expression) under different conditions.

## Supplementary Material

gkag063_Supplemental_Files

## Data Availability

The transcriptomic data generated for and analyzed in the current study are available from NCBI under the accession number BioProject PRJNA1279681. Codes used for the analyses in this study have been deposited at https://github.com/chienlinglin/Paramecium-splicing and https://doi.org/10.5281/zenodo.16736398.
