# Correction to ‘Splicing regulation and intron evolution in the short-intron ciliate model of endosymbiosis *Paramecium bursaria*’

**DOI:** 10.1093/nar/gkag163

**Published:** 2026-02-23

This is a correction to: Thi Ngan Giang Nguyen, Md Mostafa Kamal, Chien-Ling Lin, Jun-Yi Leu, Splicing regulation and intron evolution in the short-intron ciliate model of endosymbiosis *Paramecium bursaria, Nucleic Acids Research*, Volume 54, Issue 3, 10 February 2026, gkag063, https://doi.org/10.1093/nar/gkag063

Errors were introduced in Figure 5 where some minus ‘—’ symbols were converted to random characters. The original published article has been updated with a corrected figure.

These corrections do not affect the results, discussion or conclusions presented in the article. The publisher apologises for these errors.

