# Supplementary material for: Splicing regulation and intron evolution in the short-intron ciliate model of endosymbiosis Paramecium bursaria
Source: Nucleic Acids Res. 2026 Feb 2;54(3):gkag063. doi: 10.1093/nar/gkag063 (PMC12862383; doi:10.1093/nar/gkag063)
Supplement: gkag063_Supplemental_Files [file gkag063_supplemental_files.zip › 230126094339_Supplementary_data.pdf]

Supplementary Figure 1

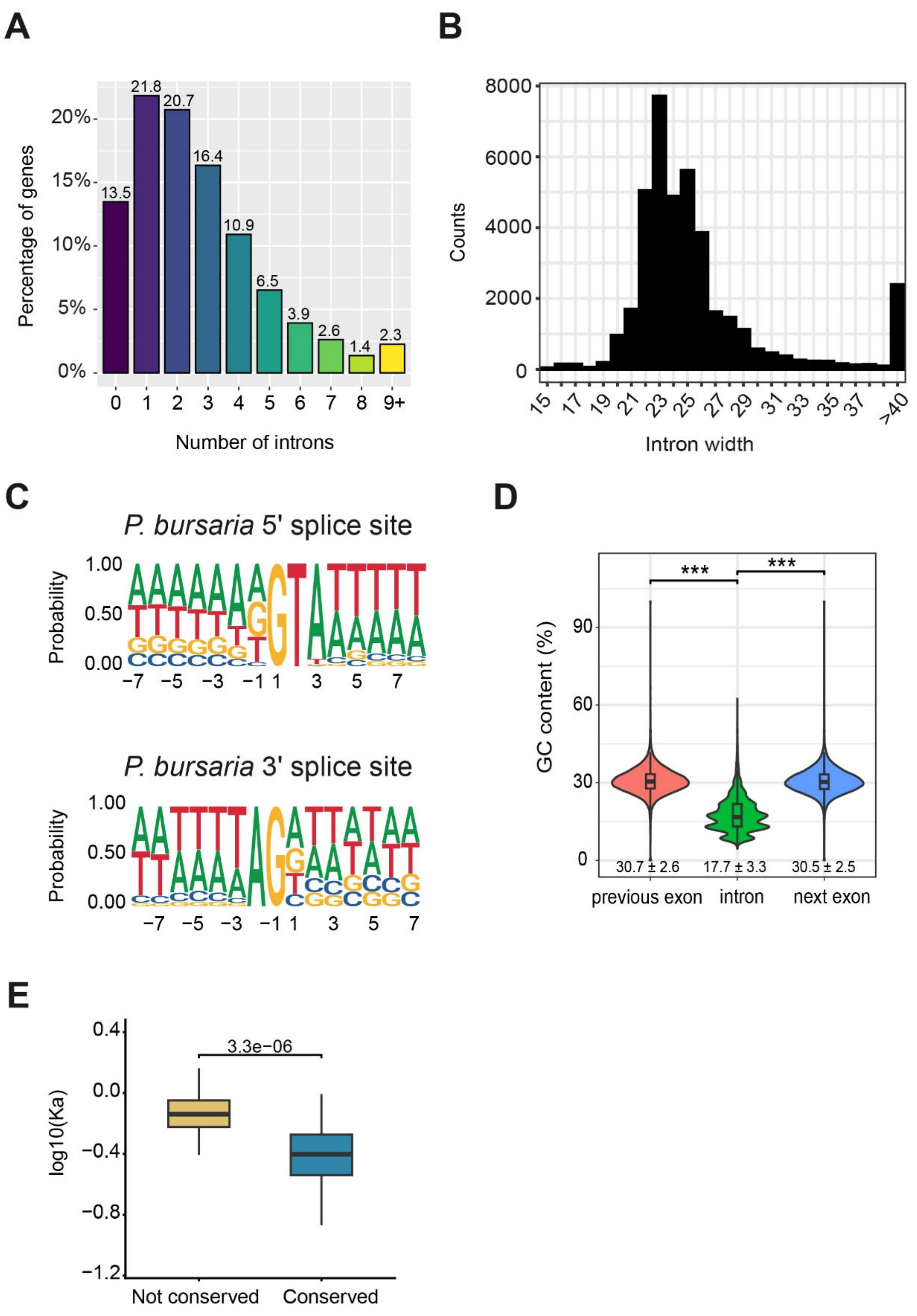

Supplementary Figure 1. Intron number, intron characteristics, and splicing efficiency in *P. bursaria*.

A. The majority of *P. bursaria* genes contain multiple introns. Barplots show the distribution of the numbers of introns in *P. bursaria* genes. B. Distribution of intron lengths (in nucleotides) in *P. bursaria* (n=39,715 introns). C. *P. bursaria* introns contain conserved 5' and 3' splice sites. Sequence logos of the 5' splice site and 3' splice site were generated using ggseqlogo R. The height of each letter represents its relative frequency at that position, with letters arranged in descending order of probability. In the 5' splice site, position 1 represents the first nucleotide of the intron. In the 3' splice site, position -1 represents the last nucleotide of the intron. D. Most introns have a lower GC content than flanking exons. Boxplots of the GC content distribution of introns and flanking exons in *P. bursaria.* The number below each boxplot indicates the average GC content ± standard error. ***, p-value < 0.001, Wilcoxon U test. E. Non-synonymous substitution rate (Ka) analysis of variably detected genes among ciliates, related to Figure 2. P-value was determined by Mann-Whitney U test.

Supplementary Figure 2

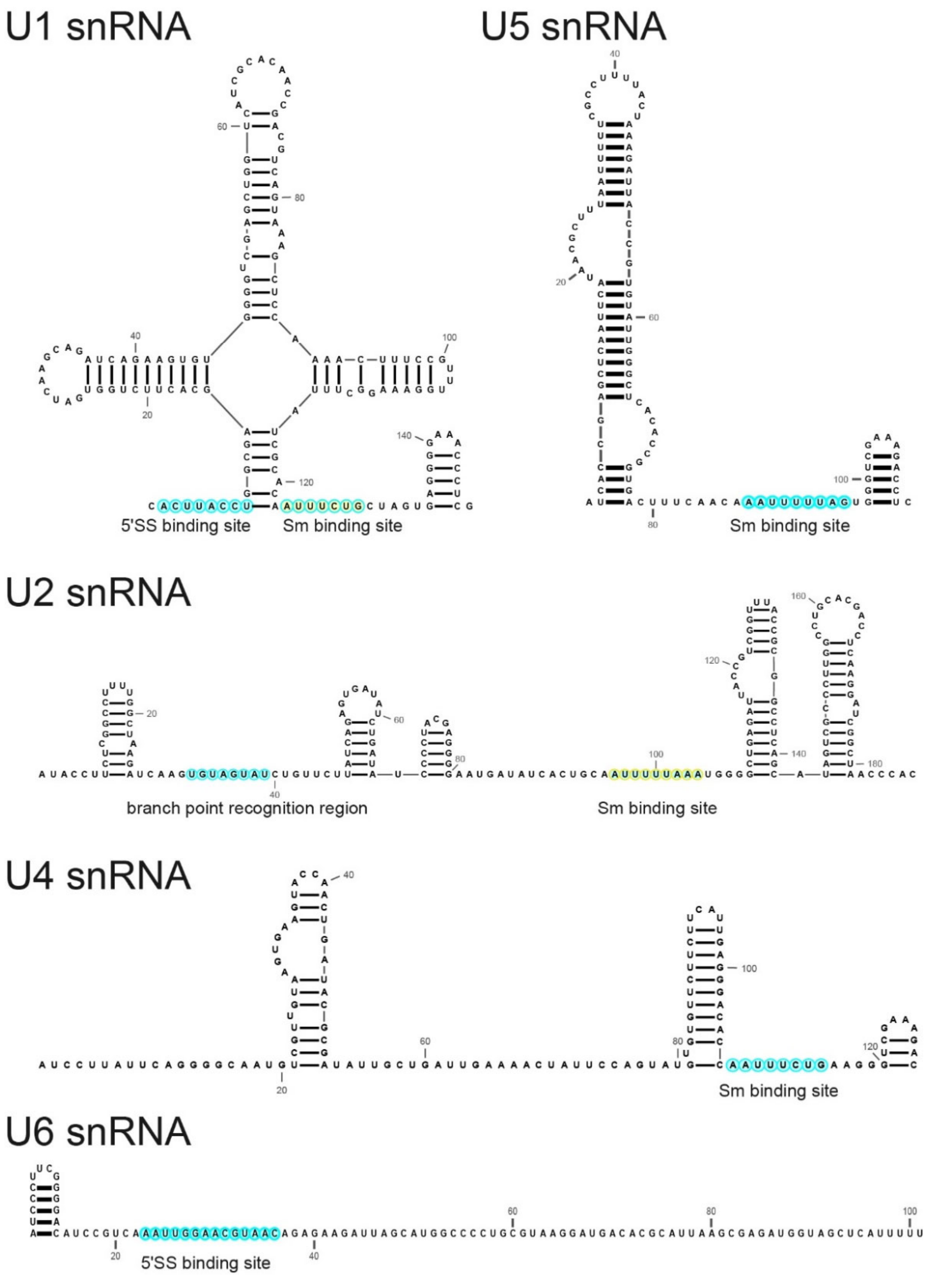

Supplementary Figure 2. Sequence and secondary structure of UsnRNAs in *P. bursaria*.

Conserved U1, U2, U4, U5, and U6 snRNAs in the *P. bursaria* genome. Key functional motifs are highlighted in color.

Supplementary Figure 3

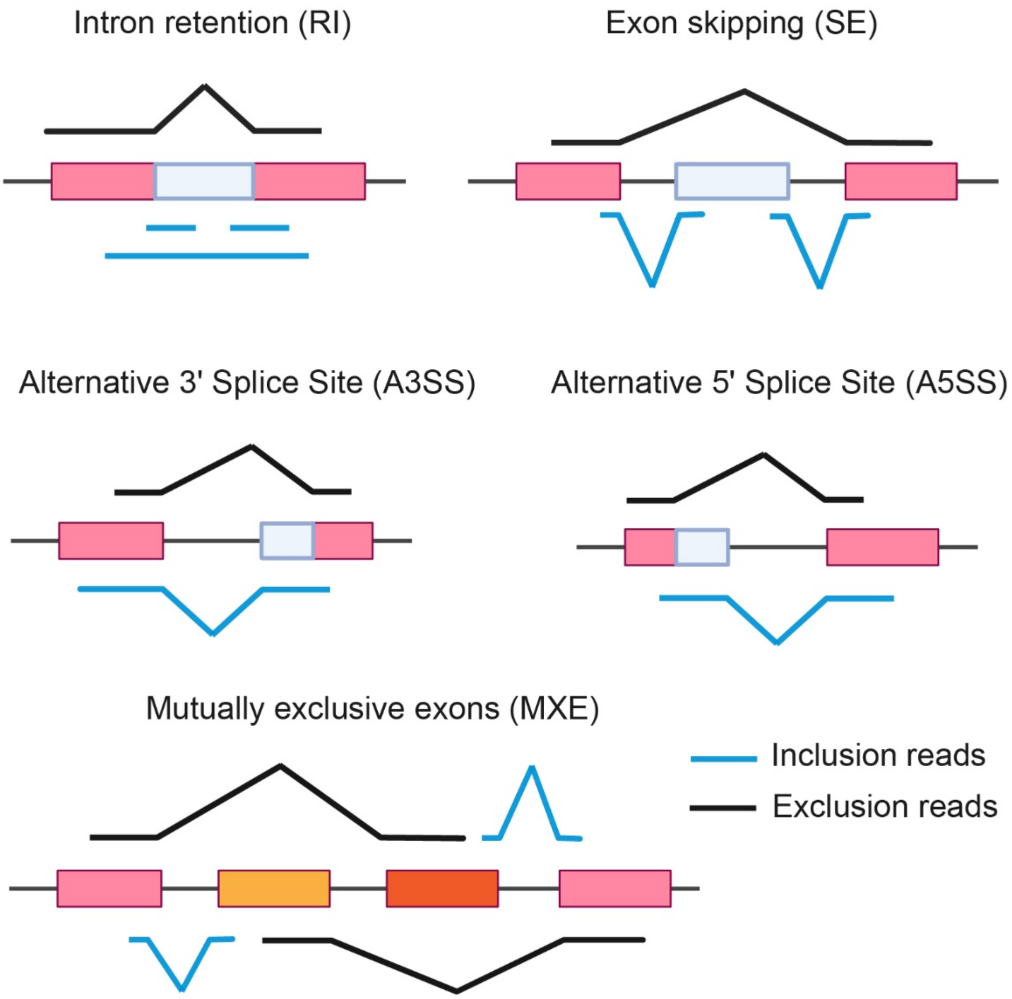

Supplementary Figure 3. Illustration of five alternative splicing events.

Supplementary Figure 4

A

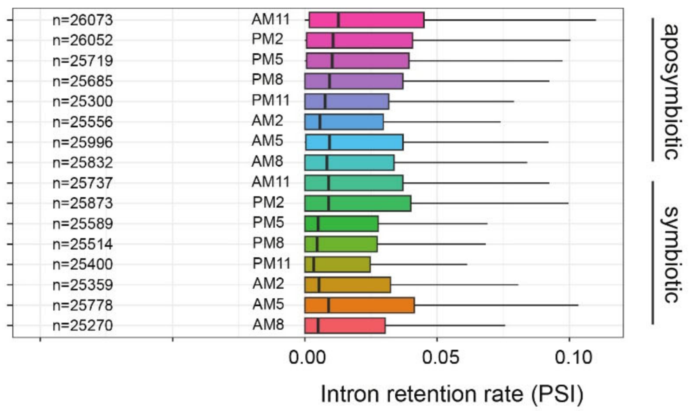

B

Pearson Correlation	3n	Non-3n	Ratio (3n/non-3n)
no correlation	196	577	0.34
positive	23	37	0.62
negative	35	124	0.28

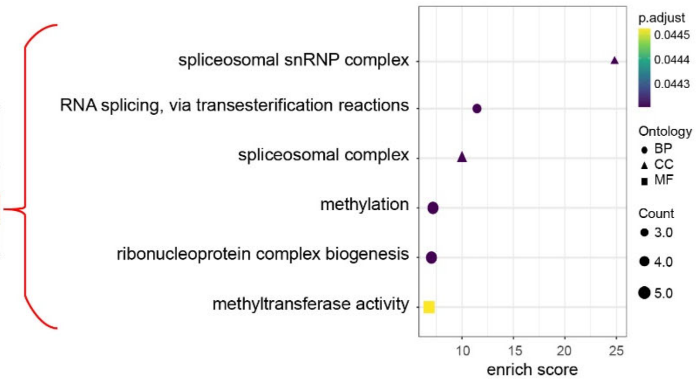

C

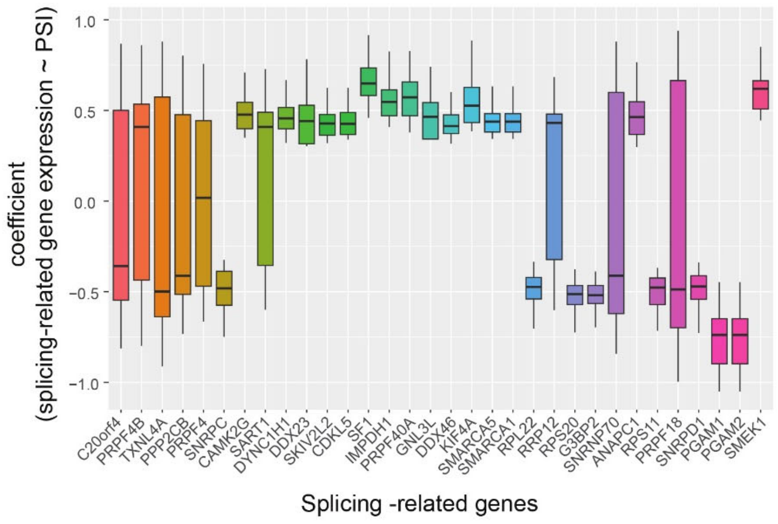

Supplementary Figure 4. Intron retention rate (PSI) distribution and the top 30 splicing-related genes based on intron numbers with significant coefficients of differential splicing.

A. Most introns are spliced efficiently in *P. bursaria.* Boxplots show the distribution of intron retention rate (PSI) across 16 samples collected from symbiotic and aposymbiotic cells. For each sample, the number of detected introns is indicated. B. Distribution of 3n and non-3n introns in relation to RNA expression (left) and gene ontology enrichment of positively correlated genes (right). BP: biological process; CC: cellular components; and MF: molecular function. C. Top 30 differentially expressed splicing-related genes that have the greatest numbers of DSIs showing significant coefficients of differential splicing. For each gene, coefficients of differential splicing were obtained by linear regression between its expression level and the PSI values of significantly associated DSI events.

Supplementary Figure 5

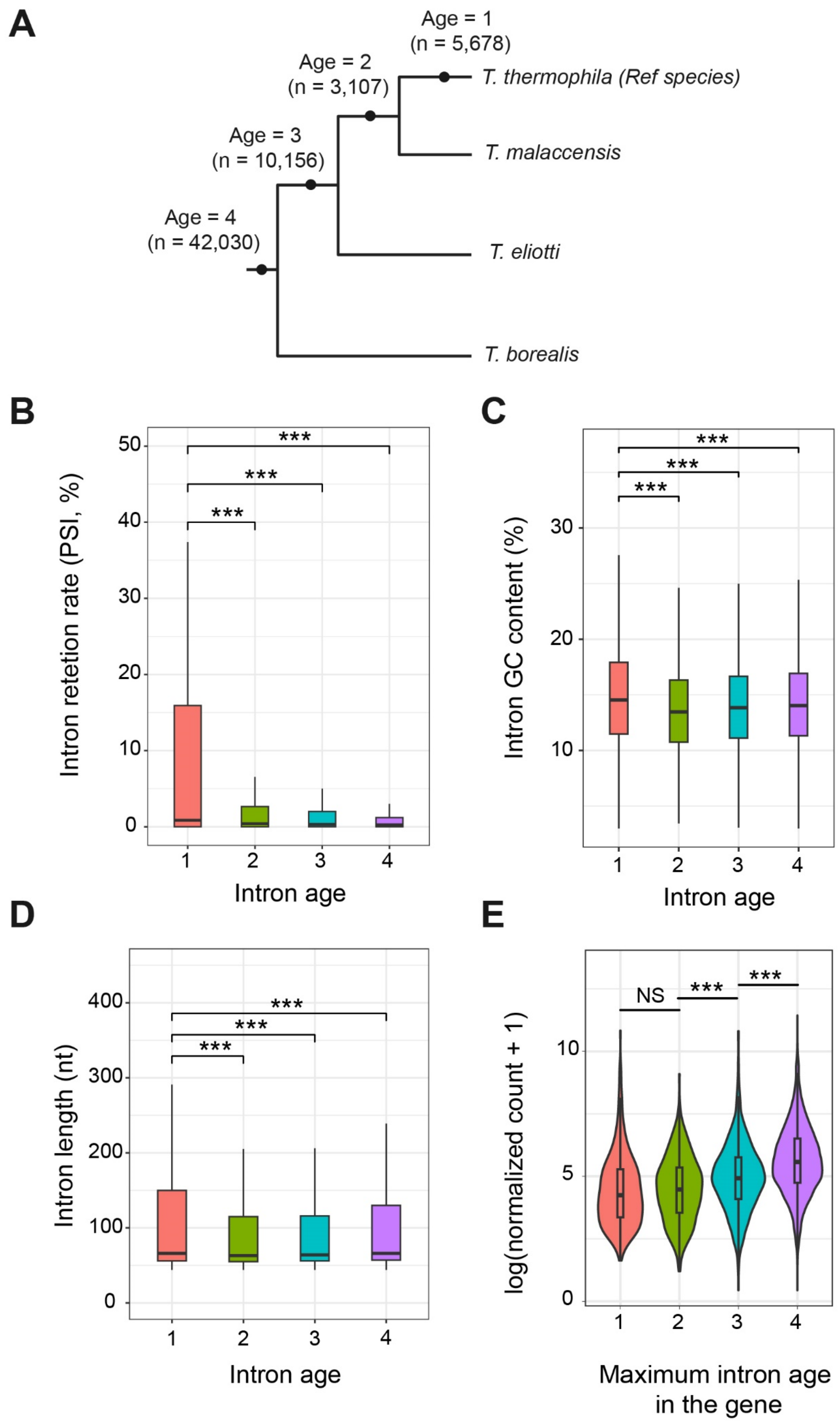

Supplementary Figure 5. Short intron evolution in *Tetrahymena* species.

A. A diagram showing the phylogenetic relationships between different *Tetrahymena* species. Introns in *T. thermophila* were assigned to different age groups based on their conservation between *T. thermophila* and the other species. Age 1 group represents the youngest introns and Age 4 group represents the oldest introns. B. Young introns exhibit higher intron retention than old introns. Boxplots show the intron retention rate for each intron age group. ***, p-value < 0.001, Mann-Whitney U test. C. Young introns have a higher GC content than old introns. Boxplots show intron GC content in each intron age group. ***, p-value < 0.001, Mann-Whitney U test. D. Young introns present a wider intron length distribution than old introns. Boxplots show intron length (in basepairs) for each intron age group. ***, p-value < 0.001, Kolmogorov-Smirnov test. E. Genes containing old introns are more strongly expressed than those solely having young introns. Boxplots show expression in each gene group based on the maximum intron age in those genes. ***, p-value < 0.001; NS, p-value > 0.05, Mann-Whitney U test.
